# A novel nanocomposite containing zinc ferrite nanoparticles embedded in carboxymethylcellulose hydrogel plus carbon nitride nanosheets with multifunctional bioactivity

**DOI:** 10.1039/d3ra02822d

**Published:** 2023-07-19

**Authors:** Fatemeh Ganjali, Mostafa Ghafori Gorab, Hooman Aghamirza Moghim Aliabadi, Saman Rahmati, Reza Ahangari Cohan, Reza Eivazzadeh-Keihan, Ali Maleki, Hossein Ghafuri, Mohammad Mahdavi

**Affiliations:** a Catalysts and Organic Synthesis Research Laboratory, Department of Chemistry, Iran University of Science and Technology Tehran 16846-13114 Iran maleki@iust.ac.ir +98-21-73021584 +98-21-73228313; b Advanced Chemical Studies Lab, Department of Chemistry, K. N. Toosi University of Technology Tehran Iran; c Protein Chemistry Laboratory, Department of Medical Biotechnology, Biotechnology Research Center, Pasteur Institute of Iran Tehran Iran; d Nanobiotechnology Department, New Technologies Research Group, Pasteur Institute of Iran Tehran Iran reza.tab_chemist@yahoo.com; e Endocrinology and Metabolism Research Center, Endocrinology and Metabolism Clinical Sciences Institute, Tehran University of Medical Sciences Tehran Iran momahdavi@sina.tums.ac.ir

## Abstract

A novel and biologically active nanobiocomposite is synthesized based on carbon nitride nanosheet (g-C_3_N_4_) based carboxymethylcellulose hydrogels with embedded zinc ferrite nanoparticles. Physical-chemical aspects, morphological properties, and their multifunctional biological properties have been considered in the process of evaluation of the synthesized structure. The hydrogels' compressive strength and compressive modulus are 1.98 ± 0.03 MPa and 3.46 ± 0.05 MPa, respectively. Regarding the biological response, it is shown that the nanobiocomposite is non-toxic and biocompatible, and hemocompatible (with Hu02 cells). In addition, the developed material offers a suitable antibacterial activity for both *Staphylococcus aureus* (*S. aureus*) and *Escherichia coli* (*E. coli*).

## Abstract 2

A novel and biologically active nanobiocomposite is synthesized based on carbon nitride nanosheet (g-C_3_N_4_) and carboxymethylcellulose hydrogels with embedded zinc ferrite nanoparticles.

## Graphical abstract

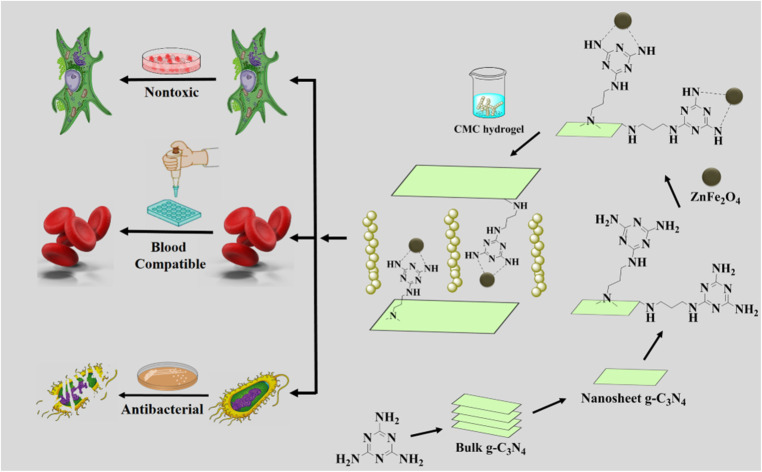

## 1. Introduction

Hydrogels represent cross-linked three-dimensional polymers, which can be obtained from natural or synthetic sources. The remarkable characteristic attributed to hydrogels is the ability to absorb water or liquids with biological properties without changing their structures.^1–3^ Hydrogels were first reported in 1960 by Wichterle and Lím.^4^ The abovementioned characteristics of hydrogels have led to their widespread use in a variety of biomedical fields, including tissue regeneration,^5,6^ contact lenses,^7^ wound dressings,^8^ expansion of stem cells,^9^ tissue engineering,^10,11^ drug delivery,^12^ functional coatings,^13^ or as antibacterial^14^ and antimicrobial^15^ materials. A variety of synthetic polymers have been used to develop hydrogels, including polyethylene glycol,^16^ polyvinyl alcohol,^17^ polyacrylamide,^18^ and polyacrylic acid.^19^ In addition, various natural polymers have also been used for this purpose, such as alginate, starch, gelatin, cellulose, chitosan, and their derivatives.^20^ Natural polymers with specific properties such as non-toxicity, biocompatibility, biodegradability, and hydrophilicity are particularly important in the biological and biomedical fields.^20–22^ Among natural polymers, cellulose and, in particular, its modified form, carboxymethylcellulose (CMC), have been the center of attention in the development of hydrogels due to natural abundance, low price, significant mechanical properties and, the simplicity of processing for the preparation of the hydrogels.^20,23–25^

Advanced hydrogels have been further developed by introducing 2D materials such as graphene analogs, allowing the tuning of specific hydrogel properties, including mechanical and electrical ones.^26–29^ In this regard, graphitic carbon nitride (g-C_3_N_4_), as a metal-free polymeric structure with two-dimensional layered morphology, has been particularly applied for various purposes, considering its chemical and thermal stability, wide surface area, biocompatibility, and non-toxicity.^30,31^ There have been growing reports on the use of g-C_3_N_4_ in areas including organic synthesis and catalyst,^32^ supercapacitors,^33^ biosensors,^34^ environmental remediation,^35^ energy,^36^ and biomedical application.^37^ Recently, g-C_3_N_4_, especially in the form of g-C_3_N_4_ nanosheets (CN), has been functionalized covalently or non-covalently by various molecules to improve its properties and applications.^38^ For example, vitamin B1 has been attached to CN by the 1,3-dibromopropane linker for the catalysis of quinoxalines.^39^ Further, melamine conjunction with g-C_3_N_4_, allows the formation of molecules with a large volume of NH_2_ groups.^40^

Also, metal nanoparticles are finding applications in catalysts,^41,42^ composites,^43–45^ sensors and microelectronics,^46^ semiconductors,^47^ and biological sciences.^48–51^ ZnFe_2_O_4_ NPs represent a biocompatible nanomaterial with excellent chemical stability, low toxicity, and potential biomedical applications.^52,53^ In addition, the ability of these nanoparticles to destroy various bacterial and microbial species causes the material to become a valuable component in biocomposites related to medical and environmental fields.^54^ ZnFe_2_O_4_ NPs have been used for the development of hybrid materials with graphene,^55^ natural and synthetic polymers,^56^ metal–organic frameworks,^57^ zeolites,^58^ and hydrogels.^59^ Magnesium hydroxide nanoparticles have been used recently as an antibacterial agent in manufacturing nanobiocomposites, including CMC hydrogels and a framework of silk fibroin for wound dressing applications.^24^

**Scheme 1:**
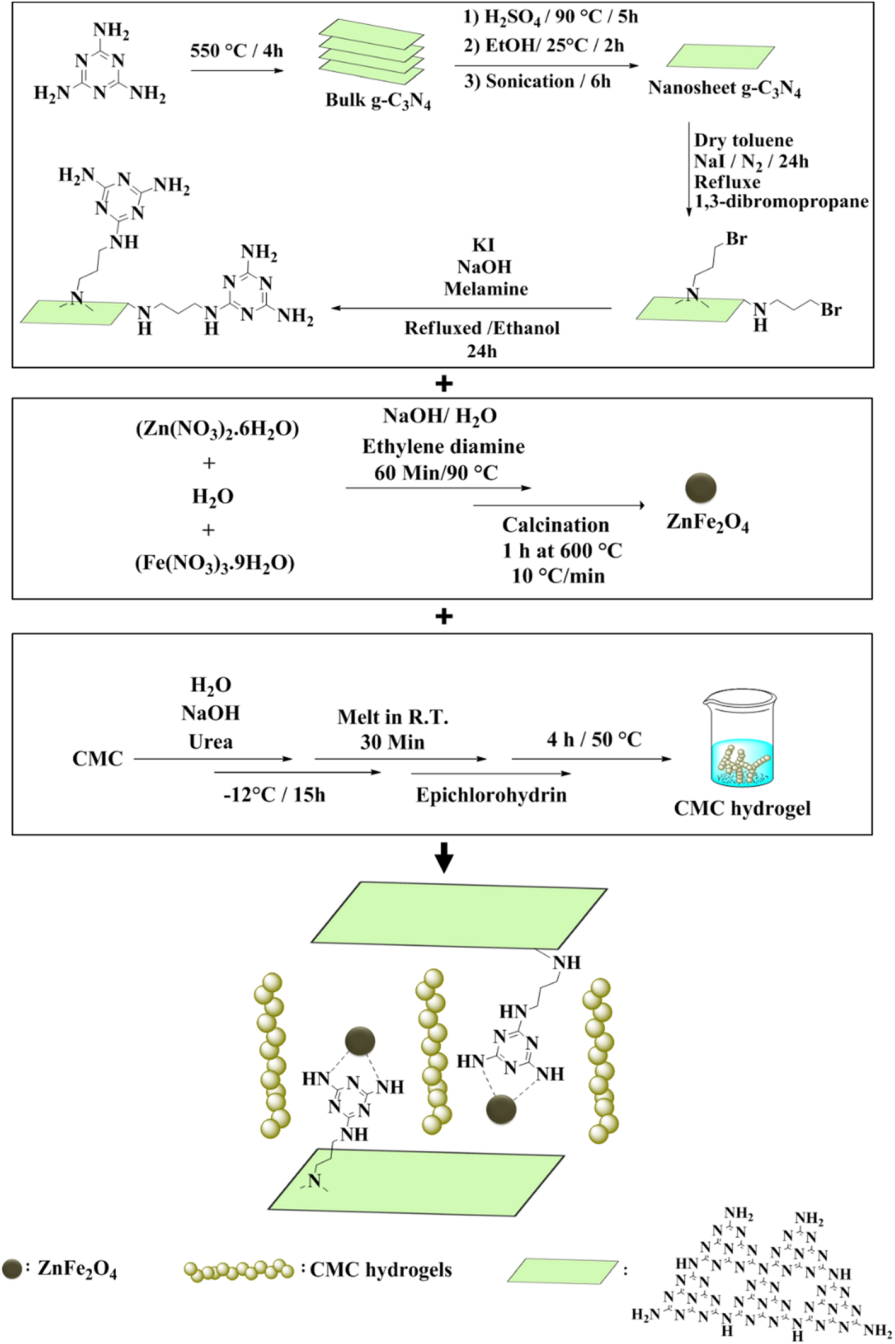
Schematic representation of the CN-Pr-Mel/ZnFe_2_O_4_/CMC hydrogel nanobiocomposite synthesis steps.

In this research, CN-Pr-Mel/ZnFe_2_O_4_/CMC hydrogel nanobiocomposites have been synthesized as a novel structure with potential application in biomedicine (Scheme 1). To synthesize this nanobiocomposite, CN is first functionalized with melamine molecules. ZnFe_2_O_4_ nanoparticles with high antibacterial potential are then added to the CN, and subsequently, CMC hydrogel is added to the structure. The structure is evaluated for its antibacterial applications.

## 2. Materials and methods

### 2.1. Materials and instruments

All materials used in this research have high purity and have been purchased from Merck and Flucka. The FT-IR spectrum of the samples is obtained with the help of KBr pellets and through an AVATAR Thermo device. EDX and FE-SEM analyses are performed using Numerix DXP-X10P and MIRA III TESCAN devices. The XRD analysis is performed in the range of 2*θ*, 5.0° to 80° with a PANalytical X-PERT-PRO MPD apparatus. Thermogravimetric analysis is carried out through an STA504 device. In this regard, the thermal stability of the sample is evaluated in the temperature range of 25 °C to 1200 °C at a temperature rate of 10 °C min^−1^ in an inert argon atmosphere. Compression mechanical properties of synthesized nanobiocomposite are evaluated according to the method presented by Eivazzadeh-Keihan *et al.*^60^ For this purpose, pieces of CN-Pr-Mel/ZnFe_2_O_4_/CMC hydrogel nanobiocomposite with approximate 1 × 1 × 1 cm dimensions are prepared. Then, compressive strength and compressive modulus are measured using a universal testing machine (SANTAM-20 model, Iran) with a load cell capacity (0.2 kN) and a crosshead rate of 0.5 mm min^−1^ at room temperature.

#### 2.1.1. Preparation of bulk g-C_3_N_4_

Bulk g-C_3_N_4_ is prepared according to the method reported by Zheng *et al.*^61^ For this purpose, white melamine powder is placed in the furnace to reach a temperature of 550 °C from room temperature in 3 hours in static air (ramp of 2.5 °C min^−1^). Then, the sample remains at 550 °C for 4 hours. The resulting yellow powder is ground for later use.

#### 2.1.2. Preparation of CN

This step is achieved based on the previous study.^62^ First, 2.0 g of the prepared bulk g-C_3_N_4_ is mixed with 40.0 mL sulfuric acid and stirred for 5 hours at 90 °C to prepare CN. After the mentioned time, bulk g-C_3_N_4_ is dissolved in sulfuric acid, forming a transparent pale yellow liquid. After cooling, the solution is diluted with 400.0 mL of ethanol and stirred at room temperature for 2 hours. By adding ethanol, the color of the solution changes to white. Then beaker contents are placed in a stationary area for two days to settle the resulting sediment. After two days, a white precipitate remains at the bottom of the container, and a clear solution is placed at the top of the beaker. Most of the clear solution is removed, and the remaining part and sediment are placed in the round-bottom flask. Afterward, the flask is placed in an ultrasonic bath for 6 hours. In the final stage, the sediment is removed using a centrifuge (5000 rpm), washed several times with water and ethanol, and placed in an oven at 80 °C for 24 hours to dry.

#### 2.1.3. Preparation of CN functionalized with 1,3-dibromopropane (CN-Pr-Br)

For this purpose, 1.0 g of CN ware disperses in 30.0 mL of dry toluene *via* ultrasonic bath for 30 minutes. Then, 2.02 mL of 1,3-dibromopropane and 1.0 mmol NaI are added to the dispersed solution and refluxed under nitrogen for 24 h. Finally, the resulting mixture is separated using a centrifuge (5000 rpm) and washed with ethyl acetate and ethanol several times. Finally, the resulting precipitate is dried at ambient temperature.^39^

#### 2.1.4. Preparation of CN functionalized with melamine (CN-Pr-Mel)

To functionalize CN by melamine, 0.5 g of g-C_3_N_4_ nanosheets with 1,3-dibromopropane are initially placed into a round-bottom flask, and 50.0 mL of ethanol is added to it. The resulting mixture is placed in an ultrasonic bath for one hour. In the next step, 3.5 g of melamine, 10.0 mL of 1.0 M NaOH solution, and 0.01 g of KI are added to the flask containing CN suspension, and the resulting mixture is stirred at ambient temperature for 15 minutes. Finally, the mixture is refluxed for 24 hours, and the obtained sediment is separated by centrifugation (5000 rpm), washed several times with water and ethanol, and dried at room temperature.

#### 2.1.5. Preparation of ZnFe_2_O_4_ NPs

For this purpose, two solutions must be first prepared separately. To prepare one of the solutions, 4.9 g of Zn(NO_3_)_2_ and 13.4 g of Fe(NO_3_)_3_ are dissolved in 50.0 mL of deionized H_2_O. For the second solution, 3.0 mL of 1,2-diaminoethane and 4.2 g of NaOH are dissolved in 70.0 mL of distilled water. Then, the solution containing metal nitrates is added to the first solution, and the resulting mixture is stirred for 1 hour at 90 °C. The obtained precipitate is separated by centrifugation and washed several times with water and ethanol. The nanoparticles are placed in a vacuum oven at 80 °C for 12 hours and then calcined for 1 hour in a furnace at 600 °C (ramp of 10 °C min^−1^).^63^

#### 2.1.6. Preparation of the CMC hydrogel

A mixture of NaOH, urea, and H_2_O is first prepared with a weight ratio of 7, 12, and 81 to prepare the CMC hydrogel. The mixture is stirred at room temperature until the solid components are completely dissolved in water. In the next step, 4.0 g of CMC powder (with an average MW of 250 000 Dalton and a degree of substitution of 0.75) is poured into 100.0 mL of the as-prepared solution in the previous step and stirred for 15 minutes at ambient temperature. Then, the lid of the container containing the mixture is closed and placed at −12 °C for 15 hours. After the mentioned time, the components of the container are removed from freezing temperature and placed at room temperature to be melted. In the next step and after melting, the solution is stirred for 30 minutes for a clear appearance. 10% weight of epichlorohydrin solution (ECH) as a cross-linker is poured into the blend and stirred for half an hour to produce a uniform solution. Eventually, after the mixture is passed through a 50 °C oven for 4 hours and a 70 °C freezer for one day, it is freeze-dried for 48 hours and then stored in a cool and dry place.^24^

#### 2.1.7. Preparation of CN-Pr-Mel–ZnFe_2_O_4_ nanocomposite

Initially, 1.0 g of CN-Pr-Mel is poured into a round-bottom flask, 50.0 mL of ethanol is added, and the mixture is placed in an ultrasonic bath for 30 minutes. Then 1.0 g of ZnFe_2_O_4_ nanoparticles are added to the suspension, and the mixture is stirred for 24 hours under reflux conditions. Finally, the synthesized nanocomposite is separated by centrifugation and dried at room temperature after washing with ethanol.

#### 2.1.8. Preparation of CN-Pr-Mel–ZnFe_2_O_4_–CMC hydrogel nanobiocomposite

In a 5.0 mL round-bottom flask, 1.0 g of nanocomposite and 2.0 mL of CMC hydrogel are poured and stirred at ambient temperature for 24 hours. Finally, the synthesized hydrogel is freeze-dried for 48 hours and stored in a cool and dry place. The detailed steps for preparing CN-Pr-Mel/ZnFe_2_O_4_/CMC hydrogel nanobiocomposites are displayed in the schematic diagram of Scheme 1 in the manuscript's main text.

## 3. Results and discussion

### 3.1. Preparation of the CN-Pr-Mel/ZnFe_2_O_4_/CMC hydrogel nanobiocomposite

The main steps have been carried out for preparing CN-Pr-Mel/ZnFe_2_O_4_/CMC hydrogel nanobiocomposites. As demonstrated in Scheme 1, the functionalization of exfoliated CN 2D nanosheets with melamine molecules *via* 1,3-dibromopropane (Pr) linker was accomplished. Then, the ZnFe_2_O_4_ nanoparticles, presenting an enhanced antibacterial characteristic, were prepared through a solvothermal method and added to the CN, followed by a CMC hydrogel addition.

### 3.2. Characterization of the CN-Pr-Mel/ZnFe_2_O_4_/CMC hydrogel nanobiocomposite

The main properties of the CN-Pr-Mel/ZnFe_2_O_4_/CMC hydrogels have been studied employing techniques including FT-IR, EDS, XRD, TGA, and FE-SEM. Functional groups, chemical bonds, structural elements, crystalline structure, thermal stability, and morphology are evaluated in this regard. Further, the mechanical properties of this new structure are also estimated.

#### 3.2.1. Functional groups and molecular vibration bands

**Fig. 1:**
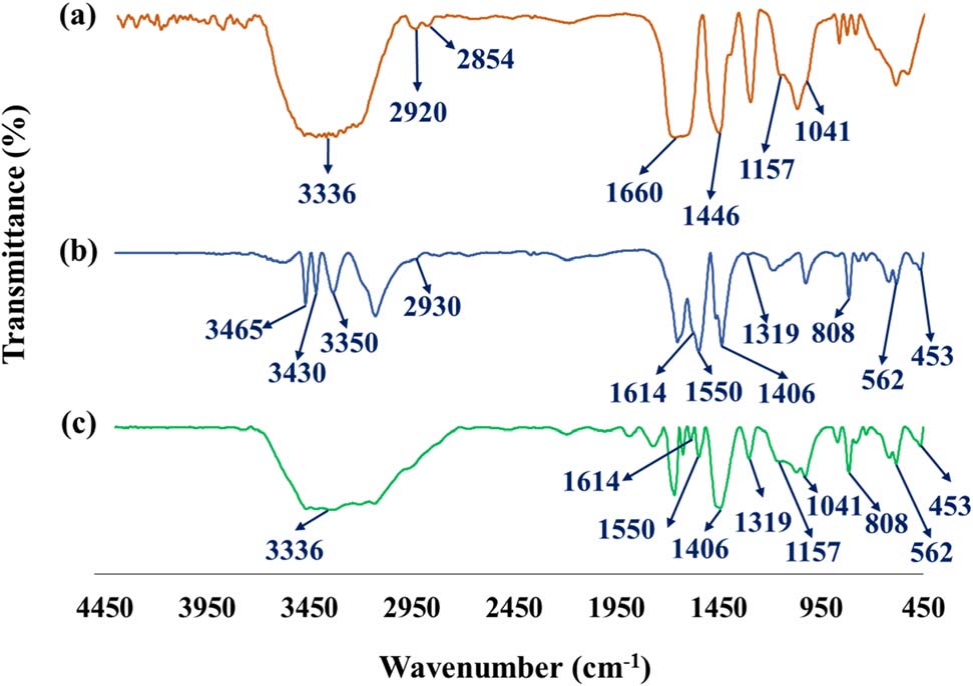
FT-IR spectrum of (a) CMC hydrogel, (b) CN-Pr-Mel/ZnFe_2_O_4_ nanocomposite, and (c) CN-Pr-Mel/ZnFe_2_O_4_/CMC hydrogel nanobiocomposite.

The formation of chemical bonds and functional groups in the preparation procedure of CN-Pr-Mel/ZnFe_2_O_4_/CMC hydrogel nanobiocomposites is evaluated by FT-IR. The FT-IR spectrum of the CMC hydrogel is shown in Fig. 1a. The broad peak observed in the 3336 cm^−1^ is related to the stretching vibrations of the OH group.^64,65^ It should be noted that the peaks observed in the regions 2854 cm^−1^, 2920 cm^−1^, 1041 cm^−1^, and 1157 cm^−1^ are related to stretching vibrations of C–H, CH_2_, C–O, and C–C groups, respectively.^24^ The C–O group is related to the CMC's pyranose ring structure and confirms the ether formation *via* its cross-linking process with epichlorohydrin. The peak observed in 1321 cm^−1^ is attributed to the hydroxyl group's bending vibration in the CMC structure.^66^ In addition, peaks of carboxyl groups after cross-linking are observed in 1446 cm^−1^ and 1660 cm^−1^ regions.^24^ The FT-IR spectrum of the CN-Pr-Mel/ZnFe_2_O_4_ nanocomposite is shown in Fig. 1b. The stretching vibrations peaks of Zn–O and Fe–O related to the structure of ZnFe_2_O_4_ nanoparticles, which are demonstrated at 453 cm^−1^ and 562 cm^−1^, respectively.^63^ The peaks detected at 1614 cm^−1^ and 1550 cm^−1^ are related to the C=N stretching vibration in the CN and melamine structure. In addition, the stretching vibration of the heterocyclic C–N can be recognized by the peaks at 1406 cm^−1^ and 1319 cm^−1^.^39,67^ The peak at 808 cm^−1^ also indicates the breathing vibration of the tri-*s*-triazine groups of the CN structure.^39^ It should be noted that concerning the CN functionalization process, the small peak in the range of 2800 cm^−1^ to 3000 cm^−1^ can be related to the stretching vibration of the C–H group of the 1,3-dibromopropane linker.^62^ Finally, peaks in the range from 3000 cm^−1^ to 3500 cm^−1^ can be related to the stretching vibration of N–H in melamine and CN.^39,67^Fig. 1c shows the FT-IR spectrum of the final nanobiocomposite, and the main peaks related to hydrogels and CN-Pr-Mel/ZnFe_2_O_4_ nanocomposite can also be observed in this spectrum, which is proof of the correct formation of the desired structure.

#### 3.2.2. Elemental composition and mapping

**Fig. 2:**
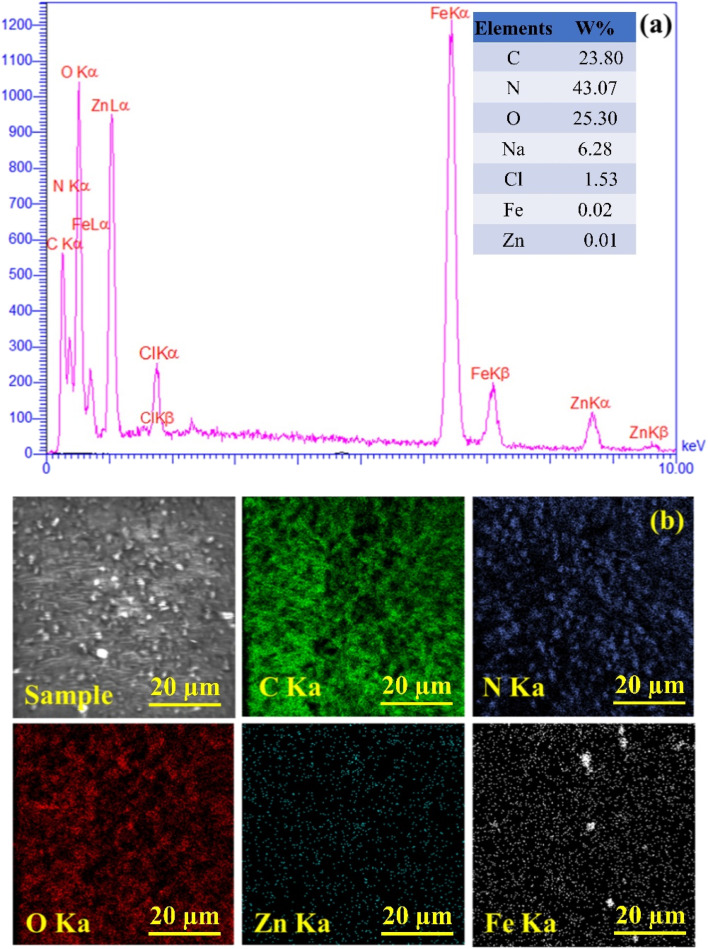
(a) EDX analysis and (b) element mapping of CN-Pr-Mel/ZnFe_2_O_4_/CMC hydrogel nanobiocomposites.

Fig. 2a shows the EDS analysis of the CN-Pr-Mel/ZnFe_2_O_4_/CMC hydrogel nanobiocomposite. All the main peaks related to the elements of the final structure, which include C, N, O, Na, Cl, Fe, and Zn, are observed in the EDS image with a weight percentage of 23.80, 43.07, 25.30, 6.28, 1.53, 0.02, and 0.01, respectively. Zinc, iron, and oxygen are related to the ZnFe_2_O_4_ nanoparticles, carbon, and nitrogen are associated with CN-Pr-Mel, and carbon and oxygen are correlated to the CMC hydrogels. In Fig. 2b, the distribution pattern of the elements in the final structure was evaluated, showing a suitable distribution in the structure with no large agglomerates or voids. However, the partial concentration of Fe refers to their aggregation during ZnFe_2_O_4_ nanoparticle formation.

#### 3.2.3. Morphological features

**Fig. 3:**
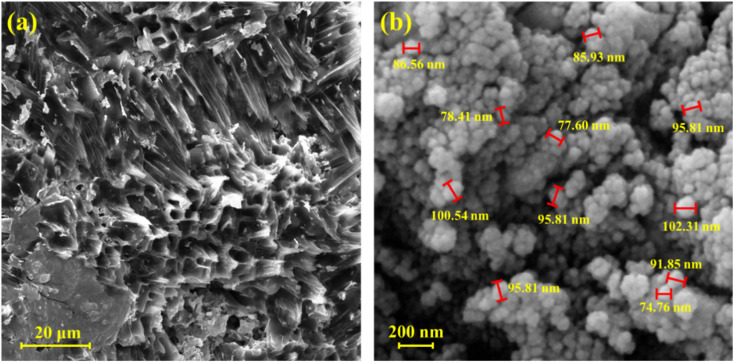
FE-SEM image of (a) CMC hydrogel and (b) CN-Pr-Mel/ZnFe_2_O_4_/CMC hydrogel nanobiocomposite.

FE-SEM images were obtained from the CMC hydrogel and the CN-Pr-Mel/ZnFe_2_O_4_/CMC hydrogel nanobiocomposite to study the morphology of the composites. The energy of the electron beam was 10.00 kV. Fig. 3a shows that the freeze-dried CMC is characterized and exhibits a completely porous structure. On the other hand, Fig. 3b shows that, after adding CN-Pr-Mel/ZnFe_2_O_4_, the morphology of the hydrogel is dominated by the nanoparticles, which are well-distributed in the hydrogel.

#### 3.2.4. Structural characteristics

**Fig. 4:**
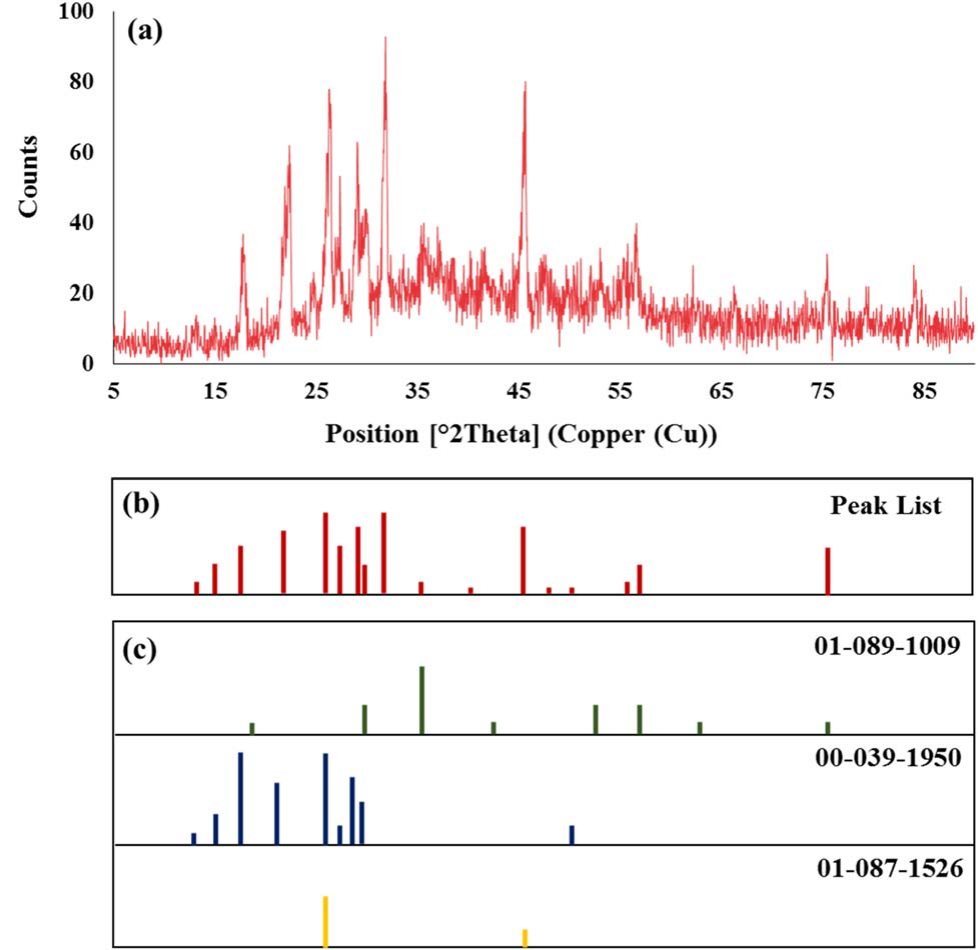
(a) XRD pattern and (b) peak list of the CN-Pr-Mel/ZnFe_2_O_4_/CMC hydrogel nanobiocomposite (c) reference of ZnFe_2_O_4_ (01-089-1009), melamine (00-039-1950) and CN (01-087-1526).

XRD analysis evaluates the crystalline structure and correct formation of CN-Pr-Mel/ZnFe_2_O_4_/CMC nanobiocomposite. All the main peaks are observed for the nanobiocomposite components with crystalline structures in 2*θ* range from 5° to 90° (Fig. 4). Accordingly, the peaks observed in the XRD pattern of the final nanobiocomposite are shown as follows; 2*θ* of 13.07°, 14.91°, 17.76°, 22.43°, 26.38°, 27.34°, 29.06°, 30.14°, 31.92°, 35.56°, 41.61°, 45.70°, 47.46°, 50.12°, 55.28°, 56.58°, 75.38°. As is shown in Fig. 4, all of these peaks are found in the references for ZnFe_2_O_4_ (JCPDS card no. 01-089-1009), melamine (JCPDS card no. 00-039-1950), and CN (JCPDS card no. 01-087-1526).^68–70^ However, they disappeared slightly after composing with CN-Pr-Mel–ZnFe_2_O_4_ nanocomposite in Scheme 1. This reduction indicates the decrease in the power of the H-bonding connection between the cross-linked cellulosic chains.^71^ The ZnFe_2_O_4_ crystals' sizes were measured by applying Scherrer's equation and compared with the reference values.^72^ In the current study, ZnFe_2_O_4_ was prepared, and their average crystal size was calculated to be about 26.49 nm using Scherrer's equation.

#### 3.2.5. Thermal stability

**Fig. 5:**
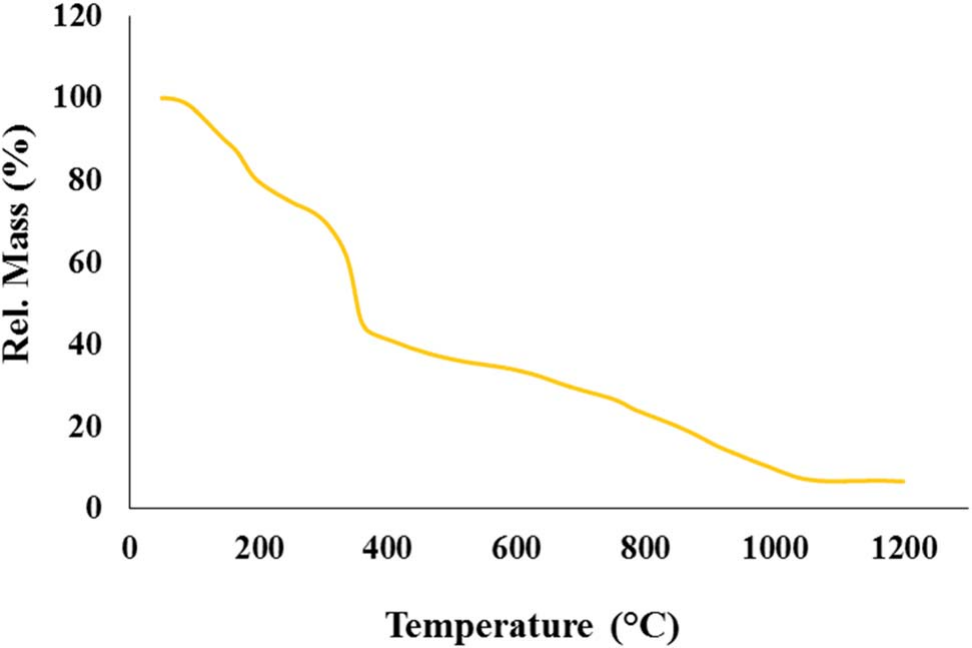
TGA curve of CN-Pr-Mel/ZnFe_2_O_4_/CMC hydrogel nanobiocomposite.

As is shown in Fig. 5, TG analysis is performed to investigate the thermal stability of the sample. This test is applicable under an argon atmosphere at a temperature rate of 10 °C min^−1^ in the range of 25 °C to 1200 °C. The first weight loss occurs from 25 to 200 °C, observed in approximately 20% of the sample weight. This value relates to releasing water and other solvents trapped in the structure.^24^ The subsequent mass reduction, which is in the range of 200 °C to 400 °C, is related to the destruction of the structure of some organic materials, including CMC and melamine.^24,73^ The organic materials destruction reduces about 40% of the sample weight. The next 40% mass drop occurs in the 400 °C to 1050 °C range, destroying the CN and ZnFe_2_O_4_.^39,63^ After 1050 °C, the weight of the sample does not change significantly, and, finally, up to 1200 °C, about 5% of the weight of the sample remains.

#### 3.2.6. Mechanical response

To evaluate the mechanical properties, two different parameters, compressive strength, and compressive modulus, are measured for the CN-Pr-Mel/ZnFe_2_O_4_/CMC hydrogels. The compressive strength and modulus are 1.98 ± 0.03 MPa and 3.46 ± 0.05 MPa, respectively.

### 3.3. Bio-application of the designed CN-Pr-Mel/ZnFe_2_O_4_/CMC hydrogel

#### 3.3.1. XTT assay

**Fig. 6:**
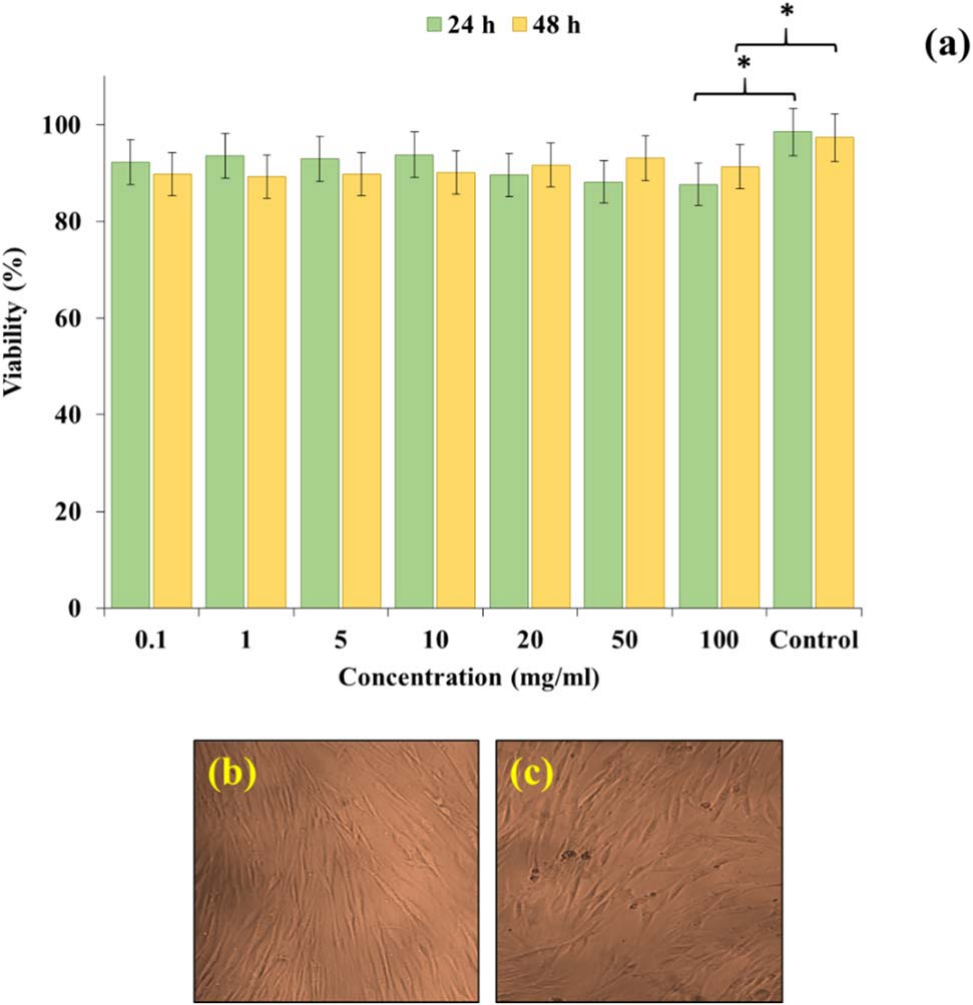
(a) XTT assay: Hu02 cells were exposed to the CN-Pr-Mel/ZnFe_2_O_4_/CMC hydrogel nanobiocomposite (* = insignificant, *P* ≥ 0.05) and (b) inverted microscopic pictures of Hu02 cells and (c) cells after contacting the nanobiocomposite.

The cell viability of the CN-Pr-Mel/ZnFe_2_O_4_/CMC hydrogel nanobiocomposites at the highest concentration (100 mg mL^−1^) is 87.6% after 24 h, which increased to 91.3% after 48 h, with no significantly different results from the control group (untreated cells). The results are the average of three independent experiments and are presented in Fig. 6a. These results indicate that this nanobiocomposite is non-toxic and biocompatible with Hu02 cells. The effect of the nanobiocomposite on cell morphology and shape is imaged with a reverse microscope, as presented in Fig. 6b and c. These Images indicate that the Hu02 cell line retains its fibroblast shape after being in contact with the CN-Pr-Mel/ZnFe_2_O_4_/CMC hydrogel.

#### 3.3.2. Hemolytic assay

**Fig. 7:**
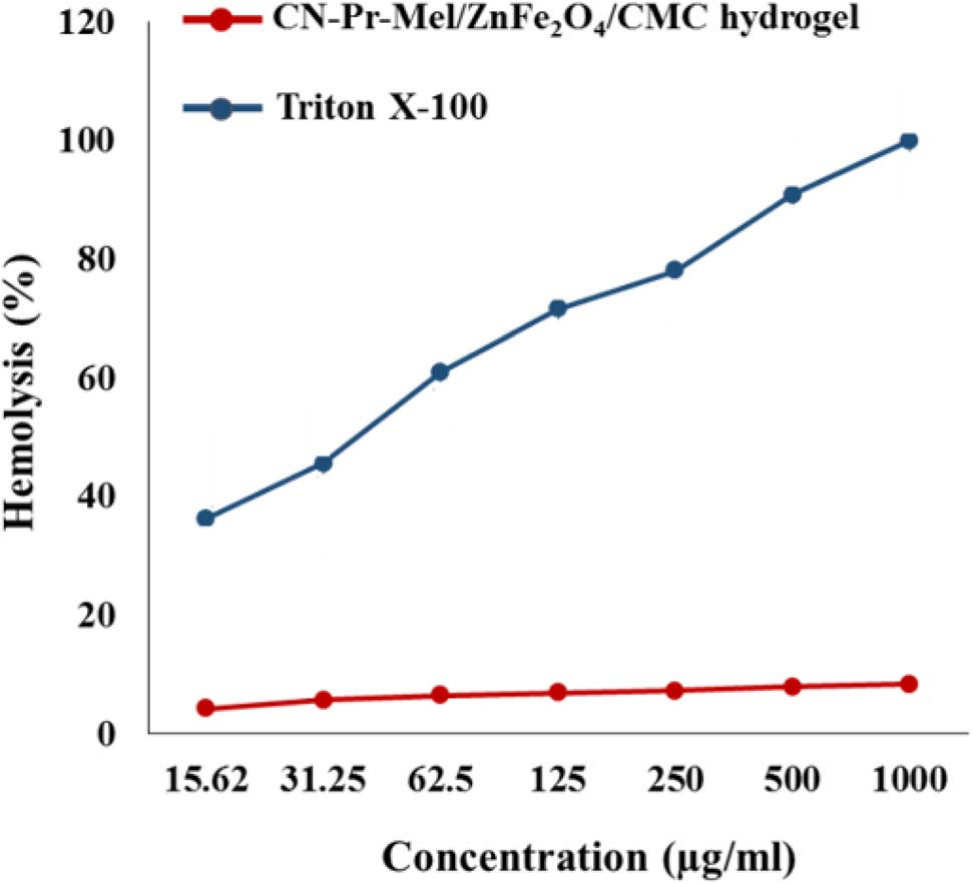
Hemolysis histogram of the positive control and CN-Pr-Mel/ZnFe_2_O_4_/CMC hydrogel nanobiocomposites (*P* ≤ 0.001).

Hemolytic assay results show that the hemolytic effect of CN-Pr-Mel/ZnFe_2_O_4_/CMC hydrogel nanobiocomposite is below 9% at 1000 μg mL^−1^. Instead, Triton X-100 is hemolyzed about 100% of RBCs at the same concentration (Fig. 7). Results are the average of three independent experiments and prove that this synthesized nanobiocomposite is hemocompatible.

#### 3.3.3. MIC and MBC determination

MICs and MBCs of CN-Pr-Mel/ZnFe_2_O_4_/CMC hydrogel nanobiocomposites and two control antibiotics (Penicillin and Streptomycin) against a Gram-positive bacteria (*Staphylococcus aureus* ATCC 25923) and a Gram-negative bacteria (Es*cherichia coli* ATCC) are determined (Table 1). Results illustrate that the MIC of the CN-Pr-Mel/ZnFe_2_O_4_/CMC hydrogel is 500 μg mL^−1^ and 1000 μg mL^−1^ for *S. aureus* and *E. coli*, respectively, demonstrating antibacterial activity.

**Table 1:** MICs in μg mL^−1^ of the CN-Pr-Mel/ZnFe_2_O_4_/CMC nanobiocomposite hydrogel against Gram-positive and Gram-negative bacteria

Agents	MIC_mean_ ± SD (MBC_mean_ ± SD) for three independent tests	
	*S. aureus*	*E. coli*
CN-Pr-Mel/ZnFe_2_O_4_/CMC hydrogel	1000 ± 1.0	500 ± 1.0
Penicillin	1.4 ± 0.1	6.8 ± 0.4
Streptomycin	12.49 ± 0.0	3.1 ± 0.6

## 4. Conclusions

CN-Pr-Mel/ZnFe_2_O_4_/CMC hydrogels have been synthesized and evaluated for biomedical applications. To synthesize this nanobiocomposite, CN is first functionalized with melamine molecules. ZnFe_2_O_4_ nanoparticles with high antibacterial potential are then added to the CN, and subsequently, CMC hydrogel is added to the structure. The application of this structure for antibacterial applications is evaluated. In biological analysis, cell viability at 100 mg mL^−1^ is 87.6% after 24 h, showing biocompatibility with Hu02 cells. Further, the hemolytic effect of the structure is below 9% at the concentration of 1000 μg mL^−1^, also compatible with blood. Concerning the antibacterial activity, the MIC of the nanobiocomposite in *S. aureus* and *E. coli* is 500 μg mL^−1^ and 1000 μg mL^−1^, respectively, demonstrating the antibacterial activity.

## Conflicts of interest

The authors listed in this article have no conflict of interest.

## Supplementary Material
